# Bayesian inference of metal oxide ultrathin film structure based on crystal truncation rod measurements

**DOI:** 10.1107/S1600576717013292

**Published:** 2017-10-20

**Authors:** Masato Anada, Yoshinori Nakanishi-Ohno, Masato Okada, Tsuyoshi Kimura, Yusuke Wakabayashi

**Affiliations:** a Graduate School of Engineering Science, Osaka University, 1-3 Machikaneyama, Toyonaka, Osaka 560-8531, Japan; b Graduate School of Arts and Sciences, The University of Tokyo, 3-8-1 Komaba, Meguro, Tokyo 153-8902, Japan; c Graduate School of Frontier Sciences, The University of Tokyo, 5-1-5 Kashiwanoha, Kashiwa, Chiba 277-8561, Japan

**Keywords:** Bayesian inference, crystal truncation rods, perovskite films, Monte Carlo

## Abstract

Monte Carlo (MC)-based refinement software to analyze the atomic arrangements of perovskite oxide ultrathin films from the crystal truncation rod intensity is developed on the basis of Bayesian inference. The advantages of the MC approach are (i) it is applicable to multi-domain structures, (ii) it provides the posterior probability of structures through Bayes’ theorem, which allows one to evaluate the uncertainty of estimated structural parameters, and (iii) one can involve any information provided by other experiments and theories. The simulated annealing procedure efficiently searches for the optimum model owing to its stochastic updates, regardless of the initial values, without being trapped by local optima. The performance of the software is examined with a five-unit-cell-thick LaAlO_3_ film fabricated on top of SrTiO_3_. The software successfully found the global optima from an initial model prepared by a small grid search calculation. The standard deviations of the atomic positions derived from a dataset taken at a second-generation synchrotron are ±0.02 Å for metal sites and ±0.03 Å for oxygen sites.

## Abstract 2

Reverse Monte Carlo software to analyze the atomic arrangements of perovskite oxide ultrathin films from the crystal truncation rod intensity is developed on the basis of Bayesian inference.

## 1. Introduction   

Perovskite oxides have been studied for a long time and still attract much attention because of their variety of conductive, electronic and magnetic properties, as well as their practical applications (Tokura & Nagaosa, 2000 ▸; Goodenough, 2001 ▸; Dagotto, 2005 ▸). In the past 15 years, extensive work has been performed on epitaxial interfaces of perovskite oxides (Ohtomo *et al.*, 2002 ▸; Ohtomo & Hwang, 2004 ▸; Nakagawa *et al.*, 2006 ▸; Hwang *et al.*, 2012 ▸; Salluzzo *et al.*, 2013 ▸; Chakhalian *et al.*, 2014 ▸; Middey *et al.*, 2016 ▸). Epitaxial interfaces allow us to study atomically ordered interfaces between two different electronic systems, such as ferromagnet/superconductor interfaces or metal/insulator interfaces. Such structures are useful to test our understanding of condensed matter physics (Chaloupka & Khaliullin, 2008 ▸) and can be used to create new electronic phases (Reyren *et al.*, 2007 ▸). For this reason, enormous effort has been made to control the oxide interface structures.

Most perovskite interfaces are fabricated by using pulsed laser deposition or molecular beam epitaxy techniques while monitoring the quality of the layer-by-later growth by reflection high-energy electron diffraction. The interfacial structure is often examined by using scanning transmission electron microscopy, which is sometimes combined with electron energy loss spectroscopy (Perna *et al.*, 2010 ▸; Cantoni *et al.*, 2012 ▸). This requires slicing the sample, which sometimes causes damage to the newly exposed surface. In addition, the sample environment is hardly controlled. The resolution of the atomic displacement is typically 0.5 Å for heavy elements, which is atomic resolution. However, when we want to study the electric polarization, which is an essential value for the physical properties of oxides, the resolution is still insufficient. For this purpose, a 0.1 Å resolution is required. Such resolution can be achieved by using surface X-ray diffraction to measure the crystal truncation rods (CTRs) arising from a sudden change in electron density at a specific plane (Robinson, 1986 ▸; Feidenhans’l, 1989 ▸). This method does not require slicing of the samples, allows tuning of the sample environment and has a resolution better than 0.1 Å. The main drawback of using this technique is the difficulty of the analysis. There are many software programs and algorithms to make the analysis easier. *ROD* (Vlieg, 2000 ▸) and its successor (Vonk, 2011 ▸) are classical examples, whose main purpose is the refinement of the surface atomic arrangement. Holographic phase retrieval methods (Takahashi *et al.*, 2001 ▸; Yacoby *et al.*, 2002 ▸) are often used to generate the initial models for the refinements (Fong *et al.*, 2005 ▸; Willmott *et al.*, 2007 ▸; Yamamoto *et al.*, 2011 ▸; Fister *et al.*, 2014 ▸). Such techniques to find a good initial model are indispensable for the interfacial structure analysis of perovskite oxides (Willmott *et al.*, 2007 ▸). Electron density analysis techniques provide flexible models, although the derivation of the standard deviations of structure parameters is not straightforward. Holographic analysis relies on the assumption that the sample surface is homogeneous, which is often untrue. Iterative reconstruction (Fung *et al.*, 2007 ▸; Björck *et al.*, 2008 ▸) shows that the true structure cannot be obtained without enforcing very strict constraints on the electron density in real space, such as positivity, atomicity, and similarity between the substrate and film structures.

**Figure 1:**
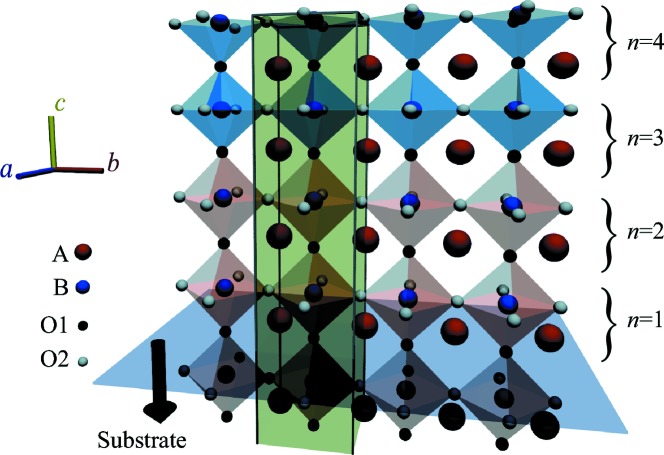
Schematic of the LaAlO_3_/SrTiO_3_ interface. The *A* site is occupied by La or Sr, and the *B* sites is occupied by Al or Ti. The blue and red octahedra represent AlO_6_ and TiO_6_, respectively.

Here, we develope a Monte Carlo (MC)-based refinement program to find reliable structural models for perovskite oxide interfaces. The method we adopt is similar to the reverse Monte Carlo method used for liquids or amorphous bodies (McGreevy & Pusztai, 1988 ▸; D’Alessandro & Cilloco, 2010 ▸; D’Alessandro, 2011 ▸). Each MC step makes a specific structural model, which can be regarded as a ‘very strict positivity and atomicity constraint in real space’. In principle, the MC technique can treat inhomogeneous surfaces, including domain structures. In addition, the MC calculation can provide the probability density of the structure model, that is, the standard deviations of structure parameters are directly provided when a set of experimental results is given. In this paper, we consider (001)-oriented LaAlO_3_/SrTiO_3_ ultrathin films. Fig. 1 ▸ presents a schematic of the film. La and Sr occupy the *A* site, and Al and Ti occupy the *B* site. Because of the assumed sample orientation, the structure is regarded as an alternating stack of *A*O and *B*O_2_ planes; the oxygen site in the *A*O plane is called the O1 site, and those in the *B*O_2_ plane are called O2 sites. In the present study, we refined the structure of a ten-unit-cell-thick region from the surface.

## 2. Theory   

### 2.1. Structure model   

The in-plane structure is assumed to have no superstructure, and the 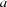 lattice parameter to be the same as that of SrTiO_3_. Now we have only two parameters per site, that is, the atomic displacements along the surface normal direction from the ideal substrate lattice 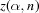 and the occupancy 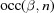, where 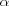 denotes *A*, *B*, O1 or O2, 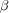 denotes La, Sr, Al, Ti, O1 or O2, and *n* denotes the layer index starting from the ideal substrate (see Fig. 1 ▸). Conditions of 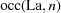 + 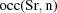 = 1 and 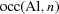 + 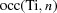 = 1 are assumed, except for *n* values close to the surface. The isotropic atomic displacement parameter 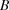 (Å^2^) was defined as being common to all atoms within the film. The total number of independent parameters *m* depends on the constraints we use, and the typical value of *m* for the present study was 60. The structural parameters of the model are expressed as 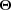 = 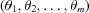 = [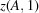, 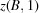, 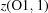, 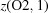, 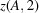,…, 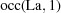, 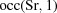, 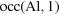, 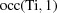, 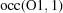, 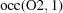,…].

### 2.2. Bayesian inference   

The experimentally observed diffraction intensity from a unit area of the sample surface at scattering vector 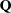 is expressed by 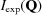. A set of CTR data is composed of the diffraction intensities at different 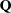, 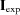 = [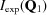, 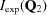,…, 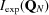], where *N* denotes the total number of measured 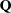 positions. Our MC software estimates the surface structure based on a posterior probability 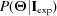, that is, a conditional probability of structural parameters under the conditions of the measured CTR data. In Bayesian inference, maximizing the posterior probability to estimate parameters is called the maximum *a posteriori* estimation. The distribution of the posterior probability reflects the uncertainty of the estimation. According to Bayes’ theorem, the posterior probability is given by
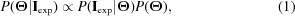
where 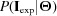 is called the likelihood function, which represents the statisical property of the measurement noise, and 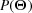 is called the prior probability, which represents prior information on the structure provided by other experiments and theories. In this paper, 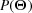 is set to a uniform distribution except in the last paragraph of §4, which means that no prior information is assumed for 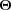. As a result, 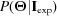 is proportional to 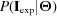 = 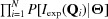. The conditional probability 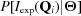 is assumed to be a Gaussian distribution with a standard deviation of 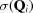:
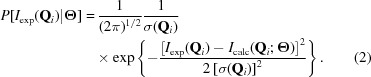
The calculated intensity for structure 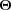 at 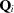 is here expressed as 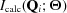 = 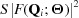, where 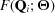 denotes the scattering amplitude for structure 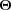 at 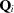 and *S* is a scale factor. Let 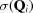 be given by 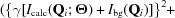 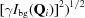 to express the Gaussian noise whose standard deviation is proportional to the intensity, which imitates the error arising from the optical misalignment. Here, 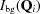 is the background intensity at 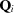. From the typical standard deviation of our CTR data at equivalent positions, we estimate the value of 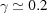.

The structure 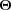 that gives the maximum value of 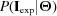 is the structure model most likely to reproduce the experimental data. For convenience, we introduce a cost function *E*(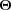) = 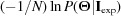, whose minimization is equivalent to maximizing a corresponding posterior distribution. Substituting equations (1) and (2) into the definition of *E*(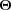), one obtains
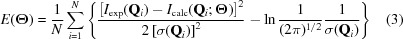
up to a constant that is independent of 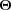.

The advantage of the MC-based technique is the flexibility of the model construction. 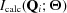 can include the effect of *B*O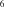 octahedral rotation or any kind of domain structure, such as domains having different film thickness, through the ordinary formula of kinematical diffraction theory. In the practical use of the MC technique, the amount of information carried by the CTR data limits the complexity of the model. 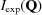 at very close 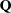 values are correlated, especially when the distance between the two points is closer than the instrumental resolution. This means that the amount of information in the CTR profile is proportional not to *N* but to the range of 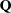 space. In this study, we selected the data step in the 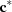 direction to be 0.02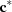, which corresponds to ∼0.2° in the 2θ angle for 16.5 keV X-rays. Thus, the resolution functions of the neighboring data points are well separated. Under these conditions, we will examine if the information derived from the CTR profiles is sufficient to obtain the global optima, by estimating both the accuracy and the precision in §3. The application to real experimental data will be presented in §4.

### 2.3. Initial model construction   

**Figure 2:**
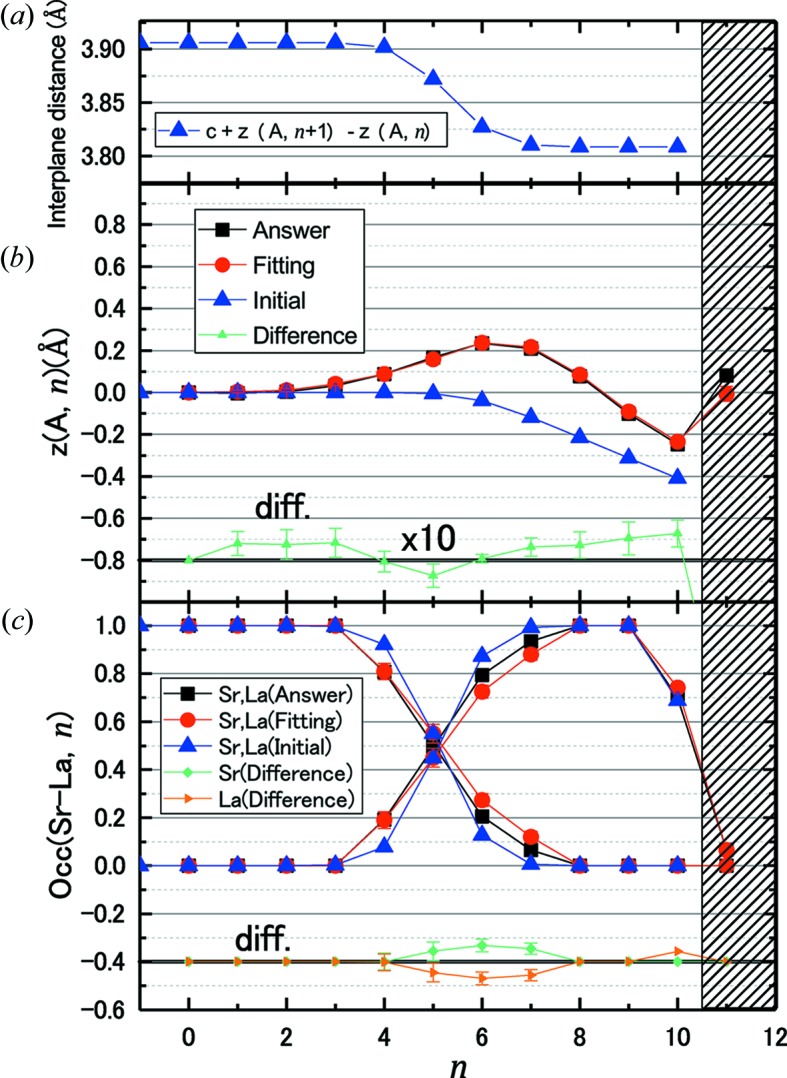
Depth dependence of (*a*) interplanar distance, (*b*) displacement of the *A* site and (*c*) occupancy of (Sr, La) of the LaAlO_3_/SrTiO_3_ heterostructure model. Error bars represent the standard deviation 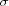 of 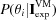. For most of the plots, the 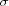 values are smaller than the symbol size. Shaded areas represent regions having occ(*A*, *n*) < 0.5. The differences between the resulting structure 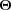 and 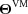 are plotted with the origin shifted to the thick horizontal lines. The difference of 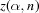 is magnified by a factor of 10.

The search for the global minimum of 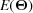 is divided into two parts: preparing initial parameters and the refinement of the parameters. The requirements of the initial model largely depend on the robustness of the refinement process. While the MC technique is robust to the initial parameters, a good initial model is preferable to achieve the model providing the global minimum of 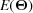. The initial values of the structural parameters are modeled by using the error functions 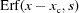, where 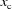 and *s* are the peak position and the width of the corresponding Gaussian function. The initial values of the interplanar distance 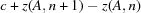 are expressed as 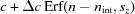, where *c* is the bulk substrate lattice parameter, 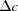 denotes the difference in the interplanar distance between the film and substrate, 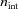 denotes the nominal interface position, and 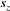 gives the sharpness of the interface. The depth profile of the initial distance 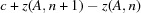 is presented in Fig. 2 ▸(*a*), and the corresponding initial 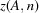 profile is shown in panel (*b*) by the blue triangles. The depth dependence of the initial value of 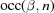 is expressed by the product of two error functions, 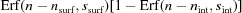 (where *n*_surf_, *s*_surf_, *s*_int_ denote the nominal surface position, the sharpness of the surface and that of the interface, respectively), as presented in Fig. 2 ▸(*c*) (blue triangles). The number of parameters is reduced to six (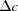, 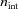, 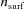, 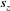, 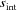, 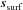), which allows us to use the grid search method to find a good initial model.

### 2.4. Monte Carlo sampling   

The refinement part of the MC calculation was performed by stochastic sampling with the Metropolis method (Metropolis *et al.*, 1953 ▸):

(1) One component of 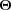 is randomly selected to be modified. The parameter is modified by a small step 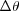 defined by a Gaussian random number with a standard deviation of 0.004 Å for 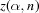 and 0.01 for 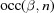.

(2) The change in 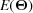 caused by the parameter modification, 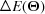 = 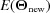 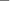 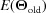, where 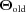 and 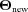 are the structure models before and after the modification, is evaluated. The probability that the modification is accepted is given by 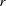, which is defined as
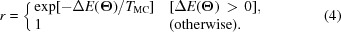
Here, 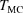 is a temperature parameter to adjust how often modifications are accepted in MC sampling. If the modification is not accepted, 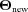 is restored to 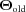.

(3) The value of 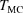 is decreased according to an exponential annealing schedule. This process is called simulated annealing (Kirkpatrick *et al.*, 1983 ▸). The number of Monte Carlo steps is typically 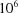
–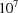. A 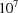 cycle iteration takes half an hour using a 4 GHz core i7 CPU with single-core calculation.

(4) After we have constructed a structural model that gives a satisfactory small value of 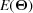, 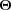 is recorded during the MC calculation with a constant temperature 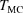 = 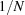, the inverse of the number of data points. This procedure generates samples from the posterior probability 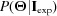 because the detailed balance condition is satisfied by the Metropolis method. Each component-wise posterior probability 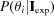 has a peak. The peak position and width are interpreted as the resulting structural parameter 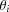 and its uncertainty, respectively. Since it evaluates the precision of structural parameters by directly sampling from their posterior probabilities, the MC technique can be applied to general cases where a strong correlation exists between the structural parameters. The values of 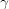 and the scale factor *S* are refined together with 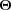.

## 3. Analysis of virtual measurement data   

**Figure 3:**
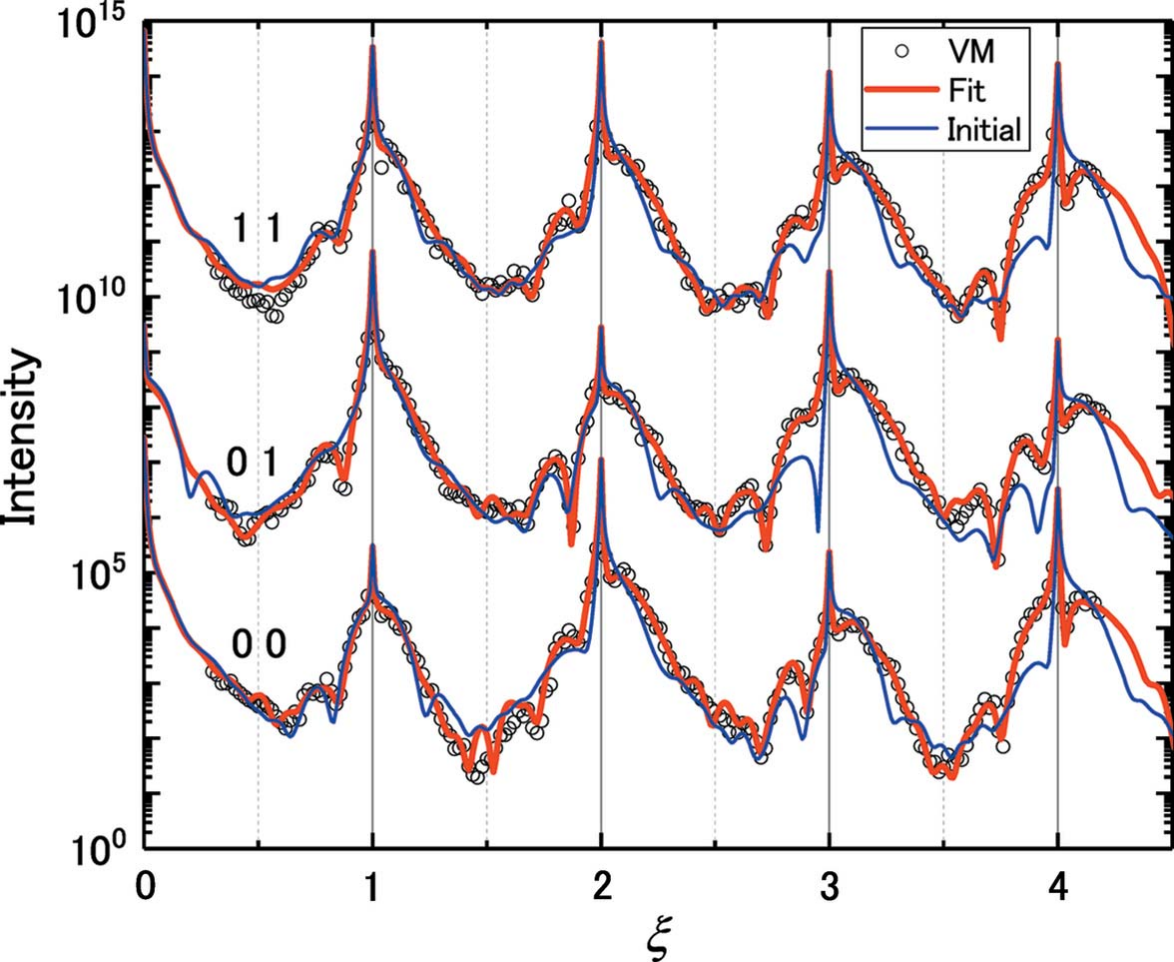
CTR intensity profile of the VM data, 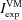 (open symbols), together with 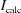 before (blue) and after (red) the MC fitting (solid curves). The three CTR rods 00ξ, 01ξ and 11ξ are shown with the scale shifted for clarity.

To evaluate the performance of the MC refinement, the software developed for this purpose was applied to artificial CTR intensity profiles calculated from the reported structure parameters of a five-unit-cell-thick LaAl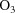 film on a TiO_2_-terminated SrTiO_3_(001) substrate (Yamamoto *et al.*, 2011 ▸); we will refer to the structure as 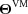. We define 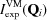 = 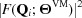 + 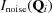. Artificial noise 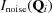 was introduced with a Gaussian distribution having a standard deviation of 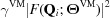, where 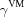 was chosen to be 0.2. Hereafter, we call 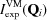 the virtual measurement (VM) data. 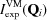 are plotted in Fig. 3 ▸. The prepared VM data were 00, 01 and 11 rods. The total number of data points 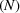 was 575. The minimum value of the cost function 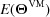 was 6.84. The initial model made with a grid search provided 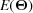 = 29.38, which corresponds to 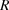 = 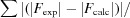 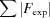 = 0.354, using the initial value of 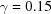. After the grid search, all variable parameters 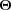 and γ were relaxed by the MC software. The MC calculation was divided into two stages by different temperature sequences. In the first temperature sequence, *z*(O1, *n*), occ(O1, *n*), *z*(O2, *n*) and occ(O2, *n*) were constrained to the corresponding values of metal ions on the same layer. The initial temperature was set to 0.5 to sample a wide parameter space. The second MC calculation with a different temperature sequence was applied to the model resulting from the first sequence by removing the constraints on *z*(O1, *n*) and *z*(O2, *n*), while the constraints on the occupancies for oxygen sites were maintained. The initial 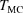 was set to 0.1 and decreased by 1% per 2000 steps. In total 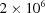 calculation steps were performed for both stages of MC calculation.

The intensity profiles calculated from the resulting structure are shown in Fig. 3 ▸ as red curves. The structure model was improved to 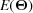 = 7.25 (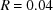), and the resulting 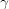 was 0.215 ± 0.002. The refined values of the structural parameters of the *A* site are presented in Fig. 2 ▸. Fig. 2 ▸(*b*) shows the depth dependence of 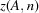. A positive displacement represents atomic movement towards the surface. Fig. 2 ▸(*c*) shows the depth dependence of 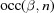. The error bars are defined by the standard deviation of each parameter.

The precision of the structure parameters is also expressed by the standard deviation of each parameter. The average value of the standard deviation of 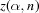 for each site 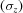 is listed in Table 1 ▸. Here, the average is taken over the sites whose occupancy is larger than 0.5. A typical value of 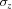 for metal sites is ±0.01 Å and that for the oxygen sites is ±0.04 Å. Standard deviations for the occupancy parameters at the interface 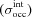 and at the surface 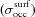 are also listed in the same table. The typical precision of the metal occupancy is 3%.

The accuracy of the structure parameters is estimated from the differences between the resulting 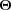 and 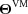, which are plotted in Figs. 2 ▸(*b*) and 2 ▸(*c*). The difference for 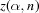 was approximately the same as the magnitude of the error bar, and the typical difference for 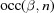 was three times larger than the standard deviation estimated by this procedure. The quantitative similarity between the accuracy and the precision suggests that the amount of information provided by the CTR profiles is well estimated by the number of data points *N* in the present case.

## 4. Analysis of experimental data   

The experimental data of LaAlO_3_/SrTiO_3_ along the 00, 01 and 11 rods with *N* = 575 (Yamamoto *et al.*, 2011 ▸) were analyzed with our MC software. The initial value of *S* was determined in advance of the MC calculation by the steepest descent method using only the data near the Bragg peaks, where the CTR intensity is rather insensitive to the detail of the surface structure model. The initial values of the other structural parameters were the same as those used for the analysis made on the VM data. The initial systematic noise scale 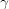 was chosen to be 0.15.

**Figure 4:**
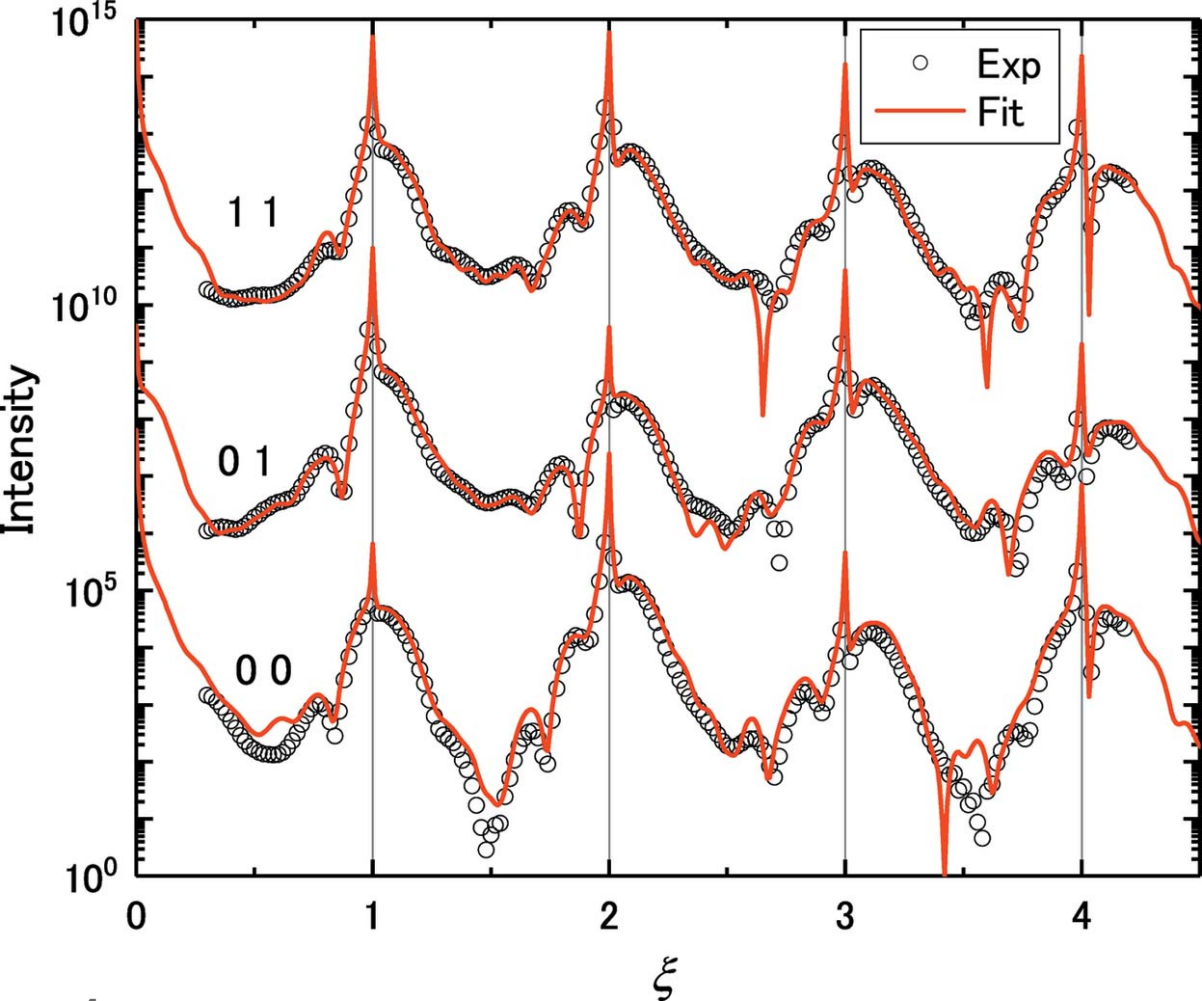
Experimental intensity profiles (open symbols) together with calculated profiles (solid curves).

**Figure 5:**
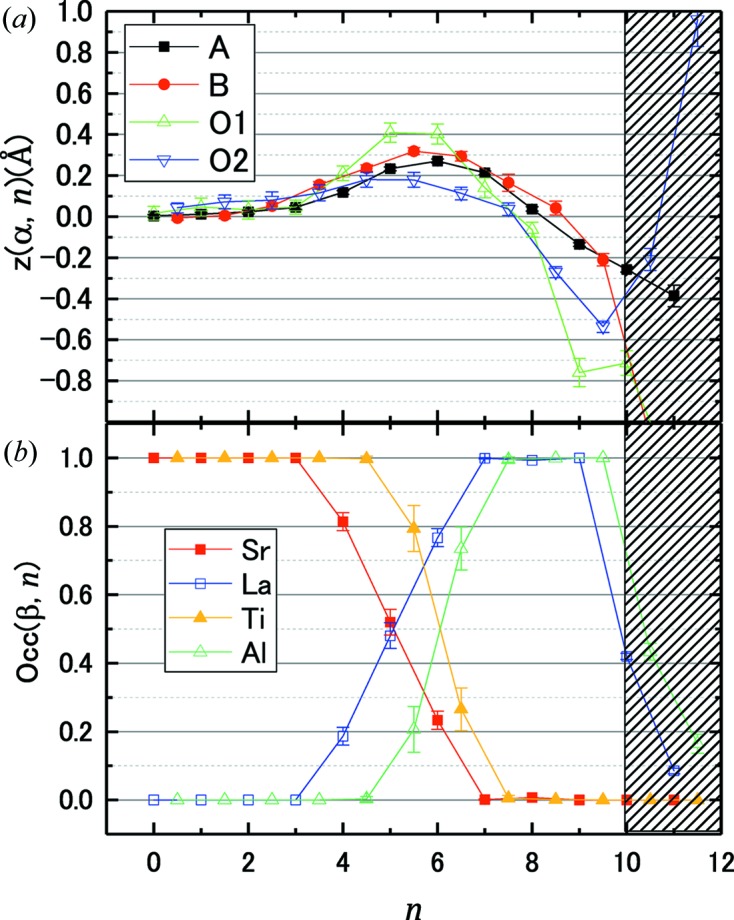
Depth dependence of (*a*) displacement and (*b*) occupancy of the LaAlO_3_/SrTiO_3_ heterostructure. The horizontal axis represents the layer index *n*. Error bars represent the standard deviation 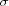 of 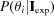. The 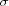 values for some parameters are smaller than the symbol size.

The initial model made with the grid search provides the value of 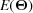 = 42.54 (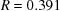). The analysis process for the MC sampling was the same as in §3, except for the refinement of the atomic displacement parameter *B* for the whole film. Fig. 4 ▸ shows the experimentally observed CTR intensity profiles together with the calculated profiles from the resulting structure. The value of 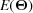 was reduced to 7.66 (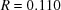). The MC refinement is proven to be robust enough for practical use of perovskite interfacial structure determination. Fig. 5 ▸(*a*) shows the depth dependence of 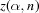. The main structural features reported by Yamamoto *et al.* (2011 ▸), namely the shift of the O atoms in SrTiO_3_ towards the surface and the lattice expansion around the interface, are reproduced in this analysis. Fig. 5 ▸(*b*) shows the depth dependence of 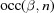. Some amount of atomic interdiffusion at the interface is visible. The average values of the standard deviation of the structural parameters are listed in Table 1 ▸, where the average was taken over the sites where the occupancy was larger than 0.5. The precision of the parameters was similar to that in the VM analysis. The *B* value for the film region was found to be 1.16 ± 0.08 Å^2^, which is about three times larger than that of the substrate atoms. We also tried to refine the *B* parameters for each atom, and found that the *B* parameters were scattered from site to site unphysically, while the other parameters were nearly unchanged. This is because the effect of each *B* parameter on the cost function is too small to refine with the present dataset.

Lastly, we present the effect of the prior probability 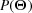. The thickness of the film in the refined structure can be defined as the total values of 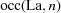 or 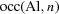, which are 4.91 ± 0.08 and 4.46 ± 0.12 in the results of the refinement presented in Fig. 5 ▸. By applying a Gaussian prior probability with an average value of 5.0 and a standard deviation of 0.1, we obtained thicknesses of 4.92 ± 0.07 and 4.74 ± 0.13 for the La and Ti sites with the cost of a slight increase in *R* value. The *R* values before and after the application of the prior probability were 0.110 and 0.111, respectively. One can define an arbitrary 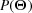, which will help to find a physically reasonable solution in a short time.

## 5. Conclusions   

We have developed MC analysis software for the CTR scattering from perovskite-type transition metal oxide interfaces. The performance of the software was demonstrated by using a five-unit-cell-thick LaAlO_3_ ultrathin film on an SrTiO_3_ substrate as an example. The precision of the structural parameters estimated from the VM data analysis was ±0.01 and ±0.04 Å for the displacement of metal and O atoms, and that for the occupancies was ±0.03. The accuracy of the structural parameters was also examined, and it was found that the accuracy was similar to the precision. Experimental data were successfully analyzed by the same procedure. We are planning to apply this method to films with some inhomogeneity in thickness.

## Figures and Tables

**Table 1:** The average value of the standard deviation of the structural parameters derived from VM and experimental data

	VM			Experimental data		
Atom	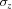 (Å)	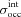	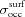	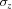 (Å)	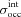	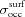
*A*	0.006	0.031	0.005	0.009	0.023	0.010
*B*	0.016	0.071	0.031	0.022	0.036	0.026
O1	0.043			0.044		
O2	0.036			0.033		
